# Varia

**Published:** 1855-07

**Authors:** 


*Varia.—Bleeding Tooth-Sockets.*—Hæmorrhage from the sockets of recently extracted teeth, are proverbially difficult of cure. Astringents, caustics, plugs, the actual cautery, cold, and various constitutional remedies, have all been known to fail, and life has been sacrificed from an exhausting hæmorrhage, proceeding from so small and easily accessible a portion of the body as the socket of a tooth. Dr. Cartwright, of New Orleans, in a late number of the Boston Journal, suggests a new plan of treatment which he has found effectual. He thinks the bleeding is encouraged by all the methods of treatment which have the effect to expand the cavity and put it upon the stretch, and that the true indication of cure is, to produce a contraction of the cavity, upon the same principle that we aim to produce uterine contraction with the view to arrest hæmorrhages from that organ. This he accomplishes by placing over the bleeding socket a pledget of raw cotton, and then applying a tourniquet over the head, with the pad placed on the outside of the cheek over the bleeding gum and the screw over the pad, acting not exactly laterally, but somewhat diagonally, so as to press upon the yielding part of the alveola process that enclosed the tooth. In his hands this remedy has never failed. The instrument is removed after wearing it for an hour or two, but kept in readiness to be reäpplied without delay in case of a recurrence of the bleeding. He relates two recent cases successfully treated on this plan, after other measures had failed.—*Memphis Med. Recorder.*

This is particularly Cartwright-ian. Fancy the pale, blanched face of a hæmorrhagic person, neatly encircled with a tourniquet, the screw forming a projecting ornament to the cheek! The simplest, neatest, and most effectual remedy for epistoxis or bleeding tooth-sockets is tr. ferri muriatis. Once, in removing a large equlis, we extracted the wisdom tooth, from the socket of which it grew. The hæmorrhage, though alarming, was instantly controlled by a pledget of cotton wet in tincture. Recently we had occasion to recommend it to a medical friend whose patient had been bleeding from a tooth-socket for twenty-four hours. He informs us that the effect was immediate and permanent. In epistoxis it should be diluted with water and snuffed up the nostrils, taking pains to carry it through to the posterior nares.

*Nicotina.*—The French smoking tobacco contains about nine per cent. of this virulent poison. In properly arranged pipes, and when the smoking is stopped before the tobacco is a l consumed, much of this substance is arrested before it reaches the mouth of the smoker; but its volatilization is aided by the presence of moisture, which accounts for the fact that smokers are more easily sickened by moist than by dry tobacco. The condensation of nicotina which takes place in the unconsumed tobacco of a pipe or segar, explains the difference in the taste of the beginning and ending of the process. The East Indians rid the tobacco smoke of most of its narcotin by making it pass thro' water, and improve its taste by the addition of cinnamon, essence of roses and a little sugar. It is advised:

1st. Not to smoke moist tobacco.

2d. To use only those pipes furnished with a condensing chamber.

3d. Not to smoke either a pipe or segar longer than till half the tobacco is consumed. And

4th. As a more certain plan for avoiding the poison—not to smoke at all. —*Ibid.*

Better still to smoke a good meerscham, which will absorb the essential oil, and if your tobacco be good, will send the smoke through to your mouth as cool and pure as the heart of the smoker could desire.

*Cupping.*—Dr. Whitfield, of Gainsville, Ala., suggests, in a letter to the editor, that it is an improvement in cupping, to stick with wax in the bottom of the cup a bit of sponge, upon which the alcohol may be dropped. All risk of burning the patient is obviated by this plan, which is adapted to all cases in which the cup can be applied vertically.—*Ibid.*

*Hydrochlorate of Ammonia.*—This salt, recommended by Watson as a remedy for hemicrania, has been found by Ebden eminently successful in neuralgia, tic-douloureux, nervous headache, toothache, clavus-hystericus, sciatica, dysmenorrhœa, and neuralgic affections generally. He directs from 25 to 35 grains to be taken at a dose, in mint water, or camphor water. Sometimes this quantity is repeated every twenty minutes until three doses are taken, but in general the second dose relieves the pain. It is advised, however, to continue the remedy at intervals of six or eight hours, to prevent a return of the disease.—*Ibid.*

The theory of the German physicians is that the sal ammoniac is analogous in its effects to calomel, especially in its action on the liver. This accords very well with the new discoveries in the physiology of the blood. Personally, we have given one or two trials to this remedy in neuralgia, with apparent success. Whatever may be its value internally, its solution is certainly one of the best gargles for common sore-throat in use, while as a discutient in external inflammations it possesses decided merits.

*Poisoning by Aconite.*—A case is reported in Boston of poisoning by swallowing one ounce of the strong tincture of aconite. Immediate vomiting ensued and continued more than an hour, severe cramp, coldness of the whole surface, and especially of the extremities. A solution was made of eight grains of iodide of potass and six grains of iodine in one quart of water. Of this a wine glass full was given, and the patient soon fell asleep. In half an hour she awoke in renewed distress; the solution was again repeated; she again fell asleep, and, in course of two hours, all formidable symptoms had disappeared. In another case of similar poisoning, great benefit was derived from laudanum, after emesis induced by ipecac.—*Ibid.*

*Nitrate of Silver.*—In the London Hospital, when this substance is required to be used in solution, the common nitric ether is preferred to water as a solvent. The common strength is eight grains to the ounce, but the solution may be made much stronger if necessary. The ether acts as a solvent of any sebaceous matter which may be on the surface, and evaporates so rapidly as to prevent spreading. A cooling sensation is at the same time produced, and several coatings may be applied in quick succession. It is much used in erysipelas, and other forms of asthenic inflammation of the skin. In addition to the several reasons given for preferring an etherial menstruum, it occurs to us, that an avoidance of the use of impure water, by which the nitrate is decomposed, is a cogent one.—*Ibid.*

*Anæsthetic Uses of Severe Cold.*—As patients now expect to have every operation performed without pain, both they and their surgeons will be glad to have an easy and agreeable means of accomplishing this, in all the common operations, unaccompanied with the dangers of chloroform. What can be less troublesome in opening an abscess, for instance, or making a cutaneous incision, than touching the skin for a moment with a brass ball that has been immersed for a few minutes in ice and salt, or a thin spoon filled with such a mixture? It is true, that in deep-seated operations such a means can only suspend the sensibility of the skin; but it is the incision of the skin which constitutes the most painful part of every operation; and if this be benumbed, a smaller, and consequently less hazardous dose of ether or chloroform than has usually been administered, would be enough to remove the sensibility of the other tissues. These deep-seated operations, however, constitute a small minority, and if the list of recorded deaths from etherization be referred to (now amounting to more than fifty) it will be found that in three-fourths of the number, complete anæsthesia might have been produced with perfect safety by cold.

M. Velpeau, who introduced anæthesia from cold into France, has, in a lecture on the subject recently reported in the Gazette des Hospitaux, expressed the doubt, whether in some operations, the hardening of tissues might not prevent their being cut with ease. I have not found this to be the case, nor does he himself allude to this disadvantage, when, in his work on diseases of the breast, he mentions that he has excised tumors after anæsthesia from cold.

The fear of reäction I have already adverted to in the prefatory observations. Instead of reäction being produced, the anæsthetic is a preventive of inflammation from the wound; and were it used for this purpose alone, it would be invaluable.—*Edinburgh Monthly Jour. of Med. Science.*

